# On mathematical modeling of the propagation of a wave ensemble within an individual axon

**DOI:** 10.3389/fncel.2023.1222785

**Published:** 2023-07-27

**Authors:** Tanel Peets, Kert Tamm, Jüri Engelbrecht

**Affiliations:** ^1^Department of Cybernetics, School of Science, Tallinn University of Technology, Tallinn, Estonia; ^2^Estonian Academy of Sciences, Tallinn, Estonia

**Keywords:** action potential, accompanying effects, laws of physics, interdisciplinary, modeling

## Abstract

The long history of studying the propagation of an action potential has revealed that an electrical signal is accompanied by mechanical and thermal effects. All these effects together generate an ensemble of waves. The consistent models of such a complex phenomenon can be derived by using properly the fundamental physical principles. In this paper, attention is paid to the analysis of concepts of continuum physics that constitute a basis for deriving the mathematical models which describe the emergence and propagation of a wave ensemble in an axon. Such studies are interdisciplinary and based on biology, physics, mathematics, and chemistry. The governing equations for the action potential together with mechanical and thermal effects are derived starting from basics: Maxwell equations, conservation of momentum, Fourier's law, etc., but modified following experimental studies in electrophysiology. Several ideas from continuum physics like external forces and internal variables can also be used in deriving the corresponding models. Some mathematical concepts used in modeling are also briefly described. A brief overview of several mathematical models is presented that allows us to analyze the present ideas of modeling. Most mathematical models deal with the propagation of signals in a healthy axon. Further analysis is needed for better modeling the pathological situations and the explanation of the influence of the structural details like the myelin sheath or the cytoskeleton in the axoplasm. The future possible trends in improving the models are envisaged.

## 1. Introduction

The propagation of signals in nerves is an extremely important chapter for understanding life. We are still far from explaining all mental and cognitive processes, but much is understood about the main physical mechanisms of electrophysiology. Besides experimental studies, mathematical modeling is an excellent tool for gaining more information about biological phenomena (Cohen, 2004). Detailed information on the importance of mathematics is given in the report of the National Research Council (2005) of the US.

Neural signaling is generally explained in terms of electrical action potentials (AP) that propagate along the nerve axon. The mathematical backbone for modeling the AP is the celebrated Hodgkin-Huxley (HH) model (Hodgkin and Huxley, 1952) and the cable equation (Rall, 1977). These models are also important for modeling dendrites and neural networks (Hines and Carnevale, 2001; Ermentrout and Terman, 2010; Bressloff, 2014; Giugliano et al., 2022).

The contemporary understanding is that signals in single axons cannot be understood in terms of electrical APs alone. It has been shown by numerous experiments that an electrical signal is accompanied by mechanical and thermal effects (Terakawa, 1985; Watanabe, 1986; Tasaki, 1988; Akkin et al., 2007; Gonzalez-Perez et al., 2016; Ling et al., 2020 for example). This means that signals in single axons is actually an ensemble of waves with several components reflecting the various coupled physical phenomena. The theoretical description of such a complex situation is described in many studies (Kaufmann, 1989; Jerusalem et al., 2019; Engelbrecht et al., 2021; Schneider, 2021).

**Figure 1:**
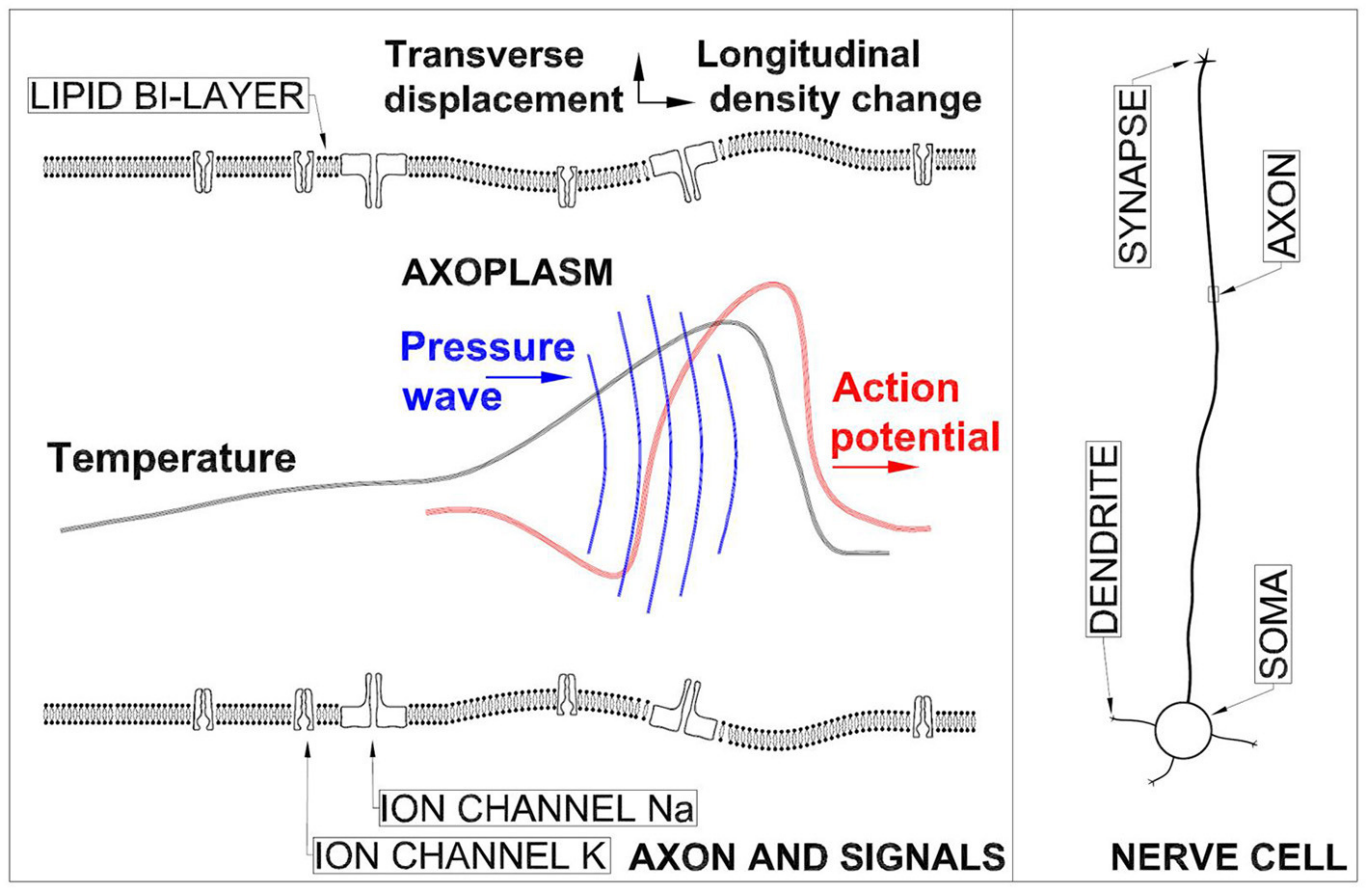
**Left panel**: Not-to-scale sketch of an axon and the signal ensemble propagation in it. **Right panel**: A sketch of a nerve cell.

An artistic sketch of a segment of the axon (which is the part of nerve cell under study here) and the signals propagating on it with the most essential structures of it can be seen in Figure 1. Axon diameter varies from a micrometer in certain nerves of the human brain to a millimeter in the giant fiber of the squid (Lodish et al., 2004). Axon length varies from a few mm up to a meter in some specialized neural cells. The lipid bilayer separating the axoplasm inside the axon from the inter-cellular medium is typically 3–4 nm in thickness and is a significant diffusion barrier enabling concentration gradient to exist across it. In Figure 1 also the Na and K channels are shown (Doyle et al., 1998; Sula et al., 2017). It must added that neurons have a rich morphology (Peng et al., 2021) and their physical forms vary greatly. The ensemble of waves is formed of an electrical AP, pressure wave in axoplasm (Terakawa, 1985), longitudinal wave in biomembrane (Griesbauer et al., 2012), transverse displacement of a bioemebrane (Gonzalez-Perez et al., 2016), and temperature changes (Tasaki and Byrne, 1992).

The analysis of the formation and propagation of an ensemble needs interdisciplinary studies involving knowledge from physics (electricity, mechanics, thermodynamics), chemistry, and mathematics (see Alvargonzález, 2011; Contera, 2019; Schneider, 2021). However, it is not sufficient just use the results of one or another branch of science, one should start by understanding the fundamental principles that establish the backbones of phenomena. This paves the way to understanding the pluralities that constitute the whole ensemble. In what follows, the attention is focused not on the formation of an AP but on the accompanying effects. These effects are important for the understanding the general energy balance during the signal propagation. A detailed analysis of complex membrane properties on the formation of the AP with emphasis on the simulation environment NEURON, is given, for example, by Hines and Carnevale (2001). NEURON is a powerful simulation tool that is not limited to single neurons, but also neural networks with complex branching morphology. It includes a catalog of different nerve cells and ion channels and the effect of ion channel distribution on formation of the AP can be analyzed. Its limitation, however, is that it does not include mechanical and thermal effects measured in many experiments.

A metaphor from the 12th century is more known from a letter by Isaac Newton dated 1675: “If I have seen further, it is by standing on the shoulders of giants”. Indeed, this special issue is devoted to Charles S. Sherrington and Edgar D. Adrian who received the Nobel prize in 1932 for their discoveries regarding the functions of neurons. It is worth noting here that the fundamental monograph by Sherrington (1906) is entitled “The Integrative Action of the Nervous System”. It means that more than 100 years ago he was thinking about mutual influence and reciprocity of effects in neurons. Contemporary studies follow this idea.

This paper aims to analyze some physical and mathematical fundamentals needed for building up models for signals in in a single axon. Section 2 is devoted to the explanation of physical effects. First, the derivation of the cable equation and its modifications has analyzed that form the basis for describing the action potential (AP). For describing the mechanical effects in axons, the wave equations need also the conservation laws as the basis. Then the importance of the Fourier law is briefly stressed. Attention is based on a special concept in continuum mechanics—the idea of internal variables that could compensate for insufficient knowledge about a physical mechanism to be modeled. Internal variables are also used by Hodgkin and Huxley (1952) for modeling the ion currents but could be used also for modeling thermal processes. Section 2 includes also some concepts of mathematics that must be properly used in modeling. In Section 3 we proceed to the general analysis based on concepts explained in previous Sections. Section 4 describes several mathematical models proposed so far for coupling the physical effects. The forward look is presented in Section 5. The main idea is to grasp better the physical internal structure of axons (myelin sheath, properties of ion channels, cytoskeleton in axoplasm etc.) to derive models able to describe not only the normal situation but also pathological cases. Conclusions are given in Section 6.

## 2. Fundamental principles as a basis for modeling

### 2.1. Physics

#### 2.1.1. Modeling of the action potential

Electrophysiology treats a signal in nerves as an electrical impulse. The classical experiments (Hodgkin and Huxley, 1952) have demonstrated that in an axon, this signal (AP) has an asymmetric shape. The axon itself is modeled as a long slender tube with a wall composed of a lipid bilayer. The governing equation for the AP is the cable equation (see Hodgkin and Huxley, 1952; Rall, 1977). To understand the application of the cable equation, we must return to basic ideas.

All the dynamic processes in nature are governed by physical laws. Considering the continuum physics, all electromagnetic processes are governed by Maxwell equations. These involve the conservation and constitutive laws for describing the charges and fluxes (Eringen and Maugin, 1990):

- conservation laws of magnetic flux and electric charge;
- constitutive laws: Ohm's law, Gauss' law, Faraday's law, Ampere's law.

Using these laws, it is possible to derive the governing equations for a one dimensional transmission line called also the telegraph equations (Lucht, 2014). In terms of potential difference (i.e., voltage) *V*(*x, t*) and current *i*(*x, t*) these equations are:


(1a) $$\begin{array}{l} \frac{\partial V}{\partial x}=-L\frac{\partial i}{\partial t}-Ri, \end{array}$$

(1b) $$\begin{array}{l} \frac{\partial i}{\partial x}=-C\frac{\partial V}{\partial t}-GV, \end{array}$$

where *L* is the inductance, *C* – capacitance per unit length, *G* – conductance per unit length and *R* – resistance per unit length. It is possible to express Equations (1) as one second order partial differential equation (PDE):


(2) $$\begin{array}{l} \frac{\partial^{2}V}{\partial x^{2}}-LC\frac{\partial^{2}V}{\partial t^{2}}-\left(LG+RC\right)\frac{\partial V}{\partial t}-RGV=0. \end{array}$$

Note that here radial and angular components of voltage are neglected.

For a process in an axon, the following assumptions are made (Bressloff, 2014):

- magnetic fields due to the movement of electric charge can be neglected;
- changes in ionic concentrations are sufficiently small so that Ohm's law holds;

Applying these assumptions, the governing equation called the cable equation, used by Hodgkin and Huxley (1952) reads:


(3) $$\begin{array}{l} \frac{\partial^{2}V}{\partial x^{2}}=RC\frac{\partial V}{\partial t}+RI, \end{array}$$

where *R* = 1/(π*a*^2^*G*), *a* is the axon radius and *I* is the ion current. In many studies, the governing cable equation is derived based on a model circuit with discrete elements (Hodgkin and Huxley, 1952; Nelson et al., 2003; Ermentrout and Terman, 2010; Bressloff, 2014).

Rall (1977) has shown that the flow of current across the membrane experiences much greater resistance than the core resistance for short length. Due to these relative resistances, steady state solutions for potential along a current exist. For transient properties of a cable also capacitance must be accounted for. Steady state and transient solutions for cable equation are dependent on the boundary and initial conditions (Rall, 1977; Giugliano et al., 2022).

According to studies made by Hodgkin and Huxley, ion current *I* depends on the opening and closing of K and Na ion channels (Hodgkin and Huxley, 1952). The decisive role in their phenomenological model is played by the phenomenological variables *n*, *m*, and *h* which lie between zero and unity. These variables are described by relaxation equations. The number of coefficients in the Hodgkin-Huxley (HH) model is rather high and needs special tuning for concrete cases. For the general analysis often a simpler model is used which relies only on one ion current. This model is called after FitzHugh and Nagumo (FHN) and in original notations reads (Nagumo et al., 1962):


(4a) $$\begin{array}{l} h\frac{\partial^{2}u}{\partial s^{2}}=\frac{1}{c}\frac{\partial u}{\partial t}-w-\left(u-\frac{u^{3}}{3}\right), \end{array}$$

(4b) $$\begin{array}{l} c\frac{\partial w}{\partial t}+bw=a-u, \end{array}$$

where *u* is the voltage, *w* the recovery current, *s* is distance along a nerve axon, and *h*, *c*, *b*, and *a* are positive coefficients. The FHN model explains the basic properties of the AP: the threshold, the asymmetric shape, and the refraction period. Although its coefficients are not related to actual experiments, this model is widely used for describing the processes in excitable media.

The cable equation and the corresponding part of the FHN model are parabolic equations because inductivity is neglected. Starting from the initial hyperbolic model like Equations (2), (3) it is possible to derive an evolution equation describing the propagation of one wave only like the celebrated Korteweg-deVries equation. In this case, leaving aside the derivation (for details see Engelbrecht, 1981), the final form in original notations is:


(5a) $$\begin{array}{l} \frac{\partial^{2}z}{\partial x\partial \xi }+f\left(z\right)\frac{\partial z}{\partial \xi }+b_{00}z=0, \end{array}$$

(5b) $$\begin{array}{l} f\left(z\right)=\mu \left(b_{0}+b_{1}+b_{2}z^{2}\right), \end{array}$$

where *z* is a properly scaled voltage, ξ is the moving coordinate (ξ = *c*_0_*t*−*x*), μ and *b*_*i*_ are constants. Velocity *c*_0_ is obtained from the hyperbolic equation, but it is not the final velocity of the pulse. Function *f*(*z*) corresponds to the FHN (one ion current). The full analysis of Equation (5) is presented by Engelbrecht (1991).

The detailed analysis of cellular mechanisms influencing the shape and the velocity of an AP in single neurons and neuron networks is given by Hines and Carnevale (2001), Clay (2005), and Debanne et al. (2011). For modeling the accompanying effects, the HH or the FHN classical models are sufficient to build up the coupling mechanisms. Note that ephaptic coupling that is an essential mechanism of interaction in sciatic nerves (Scott, 2002), modulation of axonal transmission delays (Schmidt et al., 2021), synchronous firing (Han et al., 2018), etc., are not analyzed here.

#### 2.1.2. Mechanical waves and conservation laws

All mechanical processes in the continuum are governed by conservation laws. Following Eringen (1962) and Eringen and Maugin (1990), these are:

- conservation of mass;
- conservation of momentum;
- conservation of moment of momentum;
- - conservation of energy.

In addition, the entropy inequality is used and stress-strain relation(s) are proposed. From axioms needed for a constitutive theory (Eringen and Maugin, 1990), the axiom of equipresence is of special importance: all constitutive response functionals must be considered as being dependent on the same list of constitutive variables until the contrary is deduced. The scale analysis helps with a proper choice of variables.

For modeling dynamic processes, the basic law is the conservation of momentum (in a different presentation this is the Second Newton's Law). The simplest governing equation for a wave in a continuum is the wave equation which is the 2nd order PDE and predicts the finite velocity of the process. The simple wave equation must be modified to describe dispersion, dissipation, coupling, thermal effects etc. In these cases, the higher-order terms appear in the governing equation but the basis is preserved. For an axon, one should be able to model the longitudinal wave (LW) in the biomembrane and the pressure wave (PW) in the axoplasm.

For the LW, Heimburg and Jackson (2005) have proposed a governing equation including nonlinear and dispersive effects. This equation in the improved form (Engelbrecht et al., 2015) is the following:


(6) $$\begin{array}{l} \frac{\partial^{2}u}{\partial t^{2}}=\frac{\partial }{\partial x}\left[\left(c_{0}^{2}+pu+qu^{2}\right)\frac{\partial u}{\partial x}\right]-h_{1}\frac{\partial^{4}}{\partial x^{4}}+h_{2}\frac{\partial^{4}}{\partial x^{2}\partial t^{2}}, \end{array}$$

where *u* is the density change and *p*, *q*, *h*_1_, *h*_2_ are coefficients. Note that the term with coefficient *h*_1_ models elastic effects and the term with coefficient *h*_2_ describes inertial effects of the lipids which constitute the biomembrane. Including the 4th order mixed derivatives means actually following the axiom of equipresence, which means that all effects of the same order must be taken into account simultaneously (Engelbrecht et al., 2021). This is comparable to modeling of microstructured solids (Berezovski, 2020).

For the PW, the wave equation can be used but a dissipation effect must be included. In the simplest form, it means a time derivative of the variable. Then the governing equation is:


(7) $$\begin{array}{l} \frac{\partial^{2}P}{\partial t^{2}}=c_{2}^{2}\frac{\partial^{2}P}{\partial x^{2}}-\mu_{2}\frac{\partial P}{\partial t}, \end{array}$$

where *P* is pressure in axoplasm, *c*_2_ is the characteristic velocity and μ_2_ is viscous dampening.

In terms of continuum mechanics, the biomembrane and the axoplasmic fluid can be considered like microstructured media. The main effect of the microstructure is related to dispersion. Tamm et al. (2022) have used the analogy from solid mechanics to model the longitudinal waves in the myelin sheath resulting in narrowing the profile. The question whether such an analogy could be also used for axoplasmic fluid, is open. The main difficulty is to find the physical parameters of biological “microstructured” media.

#### 2.1.3. Heat generation and physical laws

The propagation of signals along the axon is accompanied by temperature changes. Although these changes are small, the experiments have registered these changes (Abbott et al., 1958; Howarth et al., 1968; Tasaki et al., 1989). For modeling thermal effects, one should again return to basics. According to Joule's law heat is related to electric current and according to Fourier's law heat flux is related to the temperature gradient. As we are interested in temperature then the basic effect must be described by the Fourier's law:


(8) $$\begin{array}{l} q=-k\frac{\partial T}{\partial x}, \end{array}$$

where *q* is the heat flux, *T* is the temperature and constant *k* is the thermal conductivity. Consequently, the governing equation for the temperature is the heat equation


(9) $$\begin{array}{l} \frac{\partial T}{\partial t}=\frac{k}{c_{h}\rho }\frac{\partial^{2}T}{\partial x^{2}}, \end{array}$$

where *c*_*h*_ is the heat capacity and ρ – the density of the axoplasmic fluid.

#### 2.1.4. Internal variables

In continuum mechanics, it is well-known that the internal structure at the micro level may affect the process at the macro level. This influence can be described by using the concept of internal variables. Contrary to the observable variables that can be measured, internal variables are hidden and cannot be measured but the effects caused by them can give additional information about the process. A contemporary presentation of the formalism of internal variables is presented by Maugin (1990) and Maugin and Muschik (1994), illustrated by many examples in continuum physics (mechanics). In general, an internal variable is governed by an evolution equation


(10) $$\begin{array}{l} \frac{\text{d}x}{\text{d}t}=\frac{\alpha -\alpha_{0}}{\tau }, \end{array}$$

where α is an internal variable, α_0_ its equilibrium value and τ–the relaxation time.

This concept is actually used in the modeling of processes in nerves although not attributed to continuum mechanics. The celebrated HH model involves “phenomenological” variables *n*, *m*, *h* that describe changes from zero to unity. These phenomenological variables in terms of continuum mechanics can be interpreted as internal variables (Maugin and Engelbrecht, 1994).

For explaining the sources of heat generation, Abbott et al. (1958) have suggested that exothermic reactions might be taken into account but no model was proposed. Tamm et al. (2019) have proposed to use the concept of internal variables for modeling the influence of endo- and exothermic reactions to temperature changes accompanying the AP.

#### 2.1.5. External forces

As said before, the wave equations in continuum mechanics are derived by using the conservation of momentum (see Section 2.1.2). This can be expressed as force is equal to the change of momentum per change of time (see Engelbrecht et al., 2021). Indeed, in terms of stress, the governing equation is:


(11) $$\begin{array}{l} {\left(K_{ij}x_{k,j}\right)}_{,i}+\rho_{0}\left(f_{k}-A_{k}\right)=0, \end{array}$$

where *K*_*ij*_ is the Kirchoff stress tensor, *x*_*k*_ are space variables, ρ_0_ is the density of the material, *f*_*k*_ are the components of the body force and *A*_*k*_ are the components of the acceleration. Here the tensor concept (Eringen, 1962) is used, *k, i, j* = 1, 2, 3 and comma denotes the partial derivative with respect to space coordinate *x*_*k*_.

In physical terms, the additional force can be interpreted as an influence of other fields. Actually, this reflects Euler's first law: “The time rate of change of momentum of a body is equal to the sum of the forces acting on the body” (see Slattery, 1971). These forces according to Slattery (1971) are called mutual forces. In biology, one has to note the Du Bois-Reymond law (cited after Hall, 1999): “... the variation of current density, and not the absolute value of the current density at any given time, acts as a stimulus to muscle or a motor nerve". It means that for coupling, the mutual forces must be determined and they depend on changes in fields.

These concepts can be used for coupling all the effects which in our case means coupling electrical, mechanical, and thermal effects.

#### 2.1.6. Terminology

Interdisciplinary studies involve several research fields and attention should be paid to the proper usage of notions. Within the framework of modeling the signals in nerves, some remarks are needed.

The first remark concerns solitons. According to the general understanding, solitons exist in a nonlinear dispersive medium and (i) have a stable form; (ii) are localized in space; (iii) restore their speed and structure after interaction with similar entities (Ablowitz, 2011). These properties must be checked before using the notion of solitons. The iconic Korteweg-deVries equation is an excellent example of how these properties are checked. When not then in physics the notion of “solitary wave” is used. It must be stressed that the AP is not a soliton (Scott, 1999).

The second remark concerns surface waves. In physics, the surface waves propagate along the interface between different media and are well-studied in geophysics (Malischewsky, 1987). The spatial amplitude of a surface wave is confined to the vicinity of the interface, i.e., there is a strong amplitude-depth dependence reflected by the decrease of the amplitude with the distance from the interface. The idea that the longitudinal waves in a biomembrane are surface waves (El Hady and Machta, 2015) is interesting but needs special analysis from the viewpoint of classical theory.

### 2.2. Mathematics

Mathematical modeling means casting a process or a phenomenon into a mathematical representation. In biology, mathematical modeling has gained more and more attention. The features of modeling in biology are discussed in a Report by the US National Research Council (2005). Among the properties and possibilities listed in this Report are:

- models highlight basic concepts of wide applicability;
- models uncover new phenomena or concepts to explore;
- models can link what is known to what is yet unknown, etc.

Once a process is described mathematically, it needs the proper analysis. Concerning the propagation of the AP and the accompanying effects, the mathematical analysis is presented in many studies (Nelson et al., 2003; Ermentrout and Terman, 2010). Leaving aside the details of such an analysis, we mention here some important aspects that must be followed in deriving the mathematical models of nerve impulse propagation.

First, following the physical considerations (Section 2.1.5), the changes in one field will affect the other fields. In mathematical terms, it means that the changes are reflected by derivatives of variables with respect to time or space coordinates. Consequently, the forms of the coupling forces must include derivatives of variables that help to clarify the possible physical mechanisms of coupling (Engelbrecht et al., 2021).

Second, it is well-known in continuum mechanics that the transverse displacement *w* of a slender tube is proportional to the longitudinal deformation *u*_*x*_ (Engelbrecht et al., 2015). This means that if the longitudinal displacement *u* is unipolar then *w* is bipolar. The experiments where *w* has been measured in the biomembrane demonstrate that this is plausible (Terakawa, 1985; Tasaki, 1988). Note that if considering the transverse displacement as a unipolar pulse then the corresponding longitudinal displacement *u* must be a kink-type, i.e., after passing the pulse the biomembrane has a permanent change that is physically impossible.

Third, the full system of coupled equations involving the AP and accompanying effects is a system of nonlinear and linear PDEs. It involves hyperbolic (for wave-type phenomena) and parabolic (for heat-type phenomena) equations and solving this system needs numerical methods of high accuracy. For solving the coupled equations, Chen et al. (2019) have used the finite element method, and Engelbrecht et al. (2021) have used the pseudospectral method.

## 3. Concepts taken into account

Despite excellent experimental studies, there is an urgent need to build up the general overarching theory. This should be based on taking all the essential physical effects into account as mentioned by Kaufmann (1989): “electrical action potentials are inseparable from the force, displacement, temperature, entropy and other ... variables”. Andersen et al. (2009) added that there is a need “to frame a theory that incorporates all observed phenomena in one coherent and predictive theory of nerve signal propagation”. The general idea is to start by bridging the physical considerations and biological functions as stated by Schneider (2021). He stresses the importance of energy and momentum conservation and calls “to be aware of those laws right from the start and implement them in our models ...”.

Based on the brief analysis of physical principles in Section 2 (see also Engelbrecht et al., 2021) it is possible to formulate the following basic requirements for modeling:

- the signals in nerves constitute an ensemble of waves including electrical, mechanical and thermal components along and across the axis of a nerve;
- the governing equations for the components of the ensemble stem from the laws of physics including the conservation laws;
- the conservation laws of continuum physics form a consistent system which must be preserved in modifications by satisfying the axioms of the constitutive theory;
- in dynamical processes, every variation of fields acts as a stimulus (coupling force) to other fields.

The crucial stage in modeling is to understand the biological functions of processes in nerves. Numerous experiments have demonstrated the quantitative changes of variables and by combining them with mathematical formulation, some additional recommendations for modeling can be proposed:

- in the first approximation, the coupling forces are determined by first-order polynomials of gradients (space derivatives) or time derivatives; in 1D setting gradients mean changes along the axis, time derivatives across the axis;
- unipolar pulses have bi-polar derivatives and if considered as structural parts of coupling forces, are energetically stable;
- the hidden (in terms of direct measurements) processes can be described by internal variables which need additional physical parameters to be determined (equilibrium level and relaxation time);
- the mechanical waves propagating in the biomembrane (the longitudinal *u* and transverse *w* displacements) are reciprocally coupled by the so-called Rayleigh-love correction (*u*_*x*_ and *w*) meaning that the longitudinal deformation *u*_*x*_ is always coupled with a transverse displacement *w* and vice versa;
- the axiom of equipresence must be followed (for example, the elastic and inertial properties of the phospholipids must be taken into account simultaneously).

The coupling of various physical effects needs not only a clear mathematical formulation corresponding to physics but also the determination of physical parameters. This is a real challenge for experimental studies *in vivo* and *in vitro*. However, the *in silico* experiments permit to cover of a large area of quantitative physical parameters and finding suitable sets of them.

## 4. Analysis of mathematical models

The numerous experiments starting from the celebrated Hodgkin and Huxley (1952) have shown that the main carrier of information in axons is the AP with an asymmetric shape (see the overviews by Clay, 2005; Debanne et al., 2011). The wave ensemble is composed of the AP, the longitudinal (LW) wave in the biomembrane and the corresponding transverse (TW) wave, the pressure wave (PW) in the axoplasm and temperature change Θ accompanied by some biochemical changes.

The emergence and propagation of an AP under the influence of various ion channels and structural properties of an axon are intensively studied (Hines and Carnevale, 2001; Debanne et al., 2011; Goriely et al., 2015). Here we focus on the modeling of the emergence of accompanying effects. In most studies, the AP has a triggering role for all the other effects and this statement is called the Hodgkin-Huxley paradigm.

El Hady and Machta (2015) have elaborated a mathematical model based on the assumption that the potential energy is stored in the biomembrane and the kinetic energy in the axoplasmic fluid. The model takes the AP without calculations as a gaussian pulse and the attention is to determine the LW, TW, and Θ. It is stated that the mechanical modes are driven by the changes of separation across the membrane. Although the PW is not described, it is assumed that its (called the bulk flow) influence is seen as the surface waves, i.e., waves in the biomembrane. The question is that according to the general understanding (see Malischewsky, 1987), surface waves are depth-dependent and this property is not analyzed. The heat is assumed to depend on summing up the amplitudes of LW and PW, however, a detailed analysis of such an assumption is not given. The profiles of the LW, TW, and Θ correspond qualitatively to the measured ones.

A model of coupled electrical and mechanical effects based on the spring-dampers (dashpots) system is proposed by Jérusalem et al. (2014). This model describes the process in a myelinated axon and the difference in the behavior of the nodes of Ranvier and internodal regions is taken into account by the Hodgkin-Huxley model and cable theory. Tian et al. (2019) also used a similar mechanoelectrical coupled model of axons under mechanical loading.

Chen et al. (2019) proposed a coupled mechanoelectrophysiological model for axons that is based on using the flexoelectric effect. This means that changes (c.f. Section 2.1.5) of voltage field during an AP induce strain gradient fields on the axon resulting in a change of the membrane surface curvature (usually called the reverse flexoelectric effect). The AP is governed by the Hodgkin-Huxley model and cable equation, and both unmyelinated and myelinated cases are analyzed. The biomembrane is taken as an elastic or viscoelastic cylinder with a thin wall and the conservation of momentum is used for deriving the corresponding model of mechanical effects in such a cylinder. This model includes the body force due to the flexoelectric effect. The change in the axon diameter is taken into account together with the changes in the membrane capacitance and resistance. The finite-element method is used for the numerical simulation and the calculated TW has a bipolar shape. The model permits the reciprocity of electrical and mechanical effects.

A mathematical model involving all the components of the wave ensemble (AP, LW, TW, PW, Θ) is proposed by Engelbrecht et al. (2021). This model is based on the Hodgkin-Huxley paradigm and the governing equations of all the components of the ensemble except the TW are derived from the basic principles and coupled by additional forces expressing the coupling mechanisms. The TW is related to the gradient of the LW. For modeling the effects from exo- and endothermic reactions to temperature changes, the concept of internal variables is used. In this model, the main attention is on modeling the accompanying effects and therefore a simplified model for the AP - the FitzHugh-Nagumo model - is used in most calculations, although it is possible to use also the standard Hodgkin-Huxley model. The numerical simulation by the pseudospectral method demonstrated a good qualitative match with experimental studies.

A different idea for explaining the signals in nerves is proposed by Heimburg and Jackson (2005) who consider a main signal as an “electromechanical soliton". This signal is a longitudinal wave of phase transition in the biomembrane and all the other phenomena in nerves are triggered by this mechanical wave. Although this model can describe the whole process from a different viewpoint compared with the Hodgkin-Huxley model, it is not clear how the electrical signal measured by numerous experiments, is formed. It is assumed that the membrane potential is linearly proportional to the density change (Heimburg and Jackson, 2005) but this assumption does not explain the measured asymmetric shape of an AP (asymmetry means an overshoot) or the refractional overshoot (cf. the Hodgkin-Huxley or FitzHugh-Nagumo models). One should, however, note that the Heimburg-Jackson model is of great importance for explaining the dynamics in the general theory of cells where the phase transition may occur.

Schneider (2021) has studied experimentally and theoretically processes in monolayers that also permit better understand the processes in bilayers. This makes possible to unite electrical and mechanical pulses in lipids and state that the acoustic pulses in lipids have similarity to action potentials (Mussel and Schneider, 2019).

In principle, there is one more possibility to generate signals in nerves. Rvachev (2010) has assumed that the pressure wave PW in the axoplasmic fluid can trigger other phenomena in axons including the formation of an AP.

**Figure 2:**
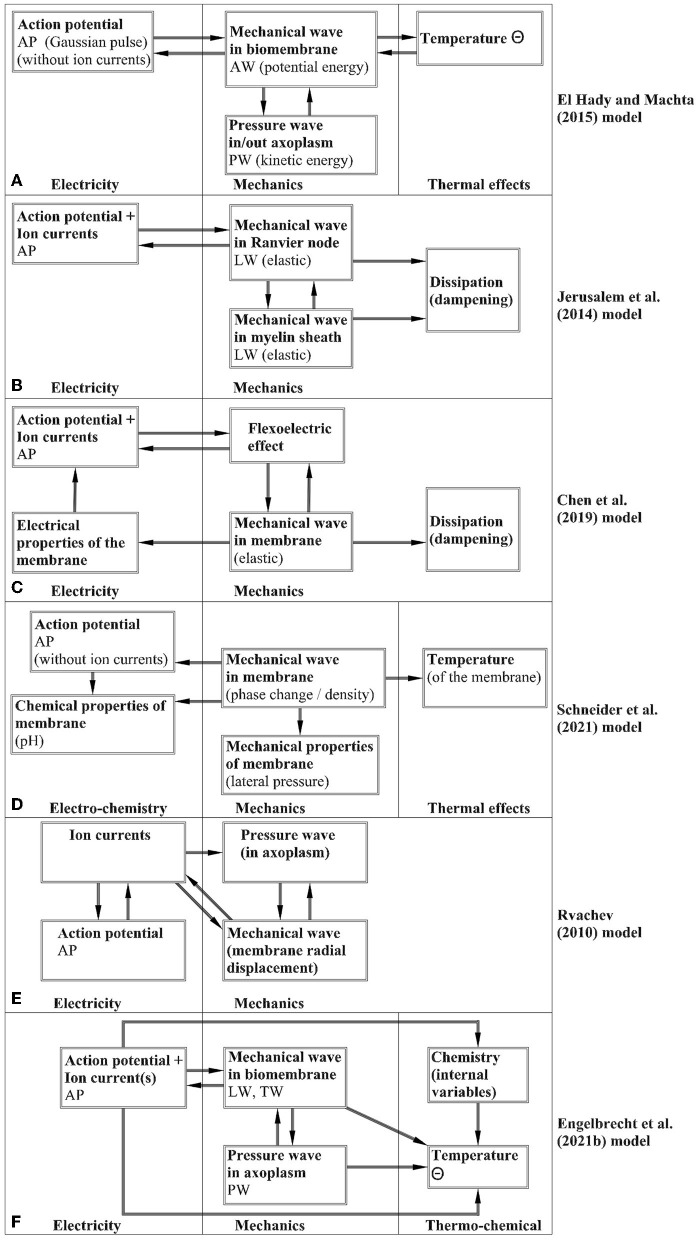
Block schemes for the noted models—**(A)** El Hady and Machta (2015); **(B)** Jérusalem et al. (2014); **(C)** Chen et al. (2019); **(D)** Schneider (2021); **(E)** Rvachev (2010); **(F)** Engelbrecht et al. (2021).

This short overview overview (see Figure 2) on recently proposed mathematical models must be enlarged by indicating also possible shortcomings in general. As noted in studies mentioned above, the presented models need to be improved following the experimental studies and better understanding the mechanisms of coupling. As far as the processes of coupling are complicated, the number of parameters to be determined is high. In overviews by Drukarch et al. (2018) and Holland et al. (2019) beside the historical facts the criticism about the phenomenological nature of several models is presented. Indeed, even the phenomenological character of the Hodgkin-Huxley model can be disputed but there is no better model which would be as widely accepted in the scientific community. The model proposed by Engelbrecht et al. (2021) uses also phenomenological relations and as mentioned by authors, needs experimental studies for determining the parameters. Actually, as noted by El Hady and Machta (2015), our knowledge about the physical properties of axons needs more data to be used in models. Consistency of governing equations are usually guaranteed by using properly the physical considerations. One of the questions raised by Drukarch et al. (2018) is the influence of the transverse displacement of the biomembrane on system properties. Chen et al. (2019) have taken it into account together with possible changes in the capacitance and the resistance of the biomembrane. Although the transverse displacement is small (in the scale of 1–2 nm), its influence on physical properties (albeit small), should be taken into account in further studies.

In modeling, attention must be paid to scales of structures and processes. The observations are summarized as follows:

- Wavelengths of the propagating signals are essential for the modeling.. If the signal duration is 2 ms and velocity is 2 m/s then the spatial length of the signal from start to finish is roughly 4 mm. If the duration is 2 ms and velocity 100 m/s then the spatial length of the signal from start to finish is roughly 20 cm. However, it must be noted that depending on the shape of the signal the spectral composition could include higher harmonics or frequency components with shorter wavelengths which could, in theory, be short enough to be sensitive toward smaller structures like ion channels or maybe even larger proteins.
- Axon diameter varies from a micrometer in certain nerves of the human brain to a millimeter in the giant fiber of the squid (Lodish et al., 2004). Axon length varies from a millimeters up to about a meter (giant fiber of the squid).
- The cycle of membrane depolarization, hyperpolarization, and return to the resting value that constitutes an action potential lasts 1–2 ms and can occur hundreds of times a second in a typical neuron (Lodish et al., 2004).
- Node of Ranvier (a structure found on myelinated axons) is typically around 1 μm in length and has a high density of ion channels (Lodish et al., 2004).
- Myelin sheath segment is typically from ≈50 to ≈300 μm in length.
- Lipid bi-layer is typically 3–4 nm in thickness.
- Mechanical transverse displacement during AP propagation is typically ≈1 nm in amplitude (Iwasa et al., 1980; Tasaki et al., 1989).
- Mammals have about 50 different small-molecule pumps (Lodish et al., 2004) (i.e., the ion channels of various kind).
- Pure phospholipid bilayers are essentially impermeable to water, but most cellular membranes contain water-channel proteins that facilitate the rapid movement of water in and out of cells (Lodish et al., 2004).
- Special transport processes involving microtubules move proteins and membranes from their sites of synthesis in the cell body down the length of the axon to the terminals (Lodish et al., 2004) (i.e., the cytoskeleton).
- The electrochemical gradients are essentially independent of the supply of ATP over the short time (Lodish et al., 2004)—meaning that AP can be propagated from a few hundred to up to a thousand times even if the ATP supply is suppressed in an axon from existing gradient across the membrane.
- AP can move down an axon without diminution at speeds up to 1 m/s (without myelin sheath) (Lodish et al., 2004). In non-myelinated neurons, the conduction velocity of an action potential is roughly proportional to the diameter of the axon (Lodish et al., 2004).
- The presence of a myelin sheath around an axon increases the velocity of impulse conduction to 10–100 m/s (Lodish et al., 2004).
- The myelin sheath is a stack of specialized plasma membrane sheets produced by a glial cell that wraps itself around the axon (Lodish et al., 2004).
- Two proteins predominate in the myelin membrane around peripheral axons: P0, which causes adjacent plasma membranes to stack tightly together, and PMP22 (Lodish et al., 2004).
- Tight junctions between the axon and the glial cell plasma membrane in the paranodal junctions immediately adjacent to the nodes may prevent diffusion of Na channels and Na/K pumps away from the nodes (Lodish et al., 2004) (although none of the noted models deal with the ionic channel distribution along the axon explicitly).

The scales listed above permit summarizing the main features of the nerve pulse propagation. Typical processes in time happen from microseconds (phase change of the lipid bi-layer in some of the described models) up to hundreds of milliseconds (temperature effects persisting after the nerve pulse has passed in some models) with most of the models predominantly focused on describing effects that are from a millisecond up to a few tens of milliseconds in time and, roughly, in phase with the main driving signal (AP or mechanical change in most of the described models). In spatial resolution the noted models fall roughly in two broad categories, first the models that are the most focused on the lipid membrane and the changes happening within (membrane thickness 3–4 nm, membrane displacement of roughly 1 nm but along the axon the signal can be from millimeters up to tens of cm in scale) and second, the models that are taking a more “continuum mechanics” approach, focusing more on the macroscopic effects in spatial scales comparable to the length of the axon and sometimes including influence of the smaller structures (like, for example, mechanosentitivity of some ion channels) in a roundabout way indirectly through parametrization of the models.

An open question is what effects described in various models are essential for a functioning of nerves and what are secondary effects accompanying the main driving signal. For example, Drukarch et al. (2018) have raised a question of membrane capacity change due to the change of axon radius. In their view this change is large enough and it must be taken into account. In the first approximation the membrane capacitance can be modeled as a plate capacitor (Heimburg, 2012) meaning that the capacitance is proportional to the surface area and inversely proportional to the -thickness of biomembrane. A mammalian axons can have diameter up to 25 μm and a change of radius by 1–2 nm (as shown in experiments) means a change of surface area by about 0.004–0.008% which is small enough to be discarded at the first approximation. For an axon with a radius of 1 μm the change of surface area would be 0.1–0.2%. On the other hand it has been shown in laboratory setting (Heimburg, 2012) that in case of a synthetic biomembrane not only area increases but also a thickness of the biomembrane shrinks. In this case a reduction of thickness in the order of 0.5 nm the capacitance would change by 14–20%. To our knowledge there have not been measurements of shrinking of the membrane thickness in actual nerve pulse propagation. However, extrapolating the result from synthetic membranes is questionable as this would also mean a change in axon length by roughly 25%. Due to the robustness of the nerve pulse modeling framework developed by Engelbrecht et al. (2021), the possible effect of changing capacitance can be easily added to the model. Chen et al. (2019) model this effect as an integral over the axon diameter and it is recalculated at every step.

Because of nonlinearities included in some of the models there might be scenarios where a small disturbances lead to large changes. That said, life is surprisingly robust and resilient, so if a given model assumes super-criticality to work or is highly sensitive toward small changes in its parameters one has to question if a given model can describe a normally operating nerve or is it more relevant for some kind of specific pathological scenario.

Finally, while mathematical modeling is certainly a powerful tool, eventually experimental data is needed to check how well reality agrees with what the models predict or assume. Unfortunately, the kinds of experiments needed to really determine what effects are important and what could be neglected as not as important are not easy to do for various reasons. Ideally, a simultaneous measurement of all the effects described in various models presented above and on several subsequent locations along the same axon would allow the determination what the noted models have gotten right and highlight any shortcoming as well.

## 5. Future research

Based on the analysis in the previous Section, several ideas could be listed for the further research which should give more information not only for a healthy axon but also improving understanding about the pathological cases is even more important.

Firstly, more data about the physical properties of an axon could better reflect the reality including the structure of an axon as well as the environment. The mathematical models described in Section 4 give the ideas for experiments that needs a good collaboration between theoreticians and experimentalists. The US National Research Council (2005) stresses the importance of mathematical models which “... are useful for formalizing intuitive understandings, even if those understandings are partial and incomplete.” It seems that a special attention should be focused on coupling mechanisms. For example, coupling forces proposed by Engelbrecht et al. (2021) involve several parameters that need quantification. These estimations must be accompanied by the scale analysis. In some studies it is assumed that the changes in the axon diameter and the corresponding changes of physical properties are small and can be neglected. In the Hodgkin-Huxley model the K and Na ion currents are taken into account (in the FitzHugh-Nagumo model just one generalized current) but the general understanding about the structure of axons and ion channels is more detailed. It is known that the structure of ion channels is complicated and may play a different role in the process of the ion pumping, especially in myelinated nerves (Arancibia-Carcamo and Attwell, 2014; Nicoletti et al., 2021). This concerns differences in the nodes of Ranvier, paranodes and juxtaparanodes that might be of importance for neuronal dysfunction. The process of formation of an AP in the axon hillock raises the question how the other accompanying effects are generated from their equilibrium states. It is known (Fields, 2014) that the initial segment of an axon controls the shape of the AP. More knowledge about emergence of the wave ensemble could cast more light on possible dysfunction of nerves.

Secondly, attention should be paid how to take the structural elements like cytoskeleton (Singh et al., 2021), molecular mechanisms (Contera, 2019), and chemical potentials (Heimburg, 2010) into account. The axoplasmic fluid can be modeled as a microstructured fluid but first the analysis of scales is needed to demonstrate that such effects influence the process. The simplest, even somewhat naive accounting of cytoskeleton influence would be just assuming that it changes viscosity of the axoplasm or acts as some kind of dampening spring on a cell membrane. However, there is also some experimental evidence [see, for example, Terakawa (1985) where removal of cytoskeleton influences an experimentally measured pressure response by an order of magnitude] that the cytoskeleton might influence the functioning and properties of an axon by a significant amount. Cytoskeleton interacting with cell membrane could change its stiffness (Franke et al., 2009) or even take part in the signal propagation. Then there is a question if the presence of a cytoskeleton could affect the local temperature changes accompanying the nerve signals. Is it continuous, could it conduct heat at different velocity along the axon than a simple diffusive heat propagation in water would indicate, does it have much different heat capacity than axoplasm, and so forth are all different questions that might change something. After all, there is a lot of chemistry going on in the metabolism of cells and chemistry could be highly sensitive toward local temperature and also to the local pH of the environment. And all that is before one considers its role in processes and metabolism of the nervous cells that are happening at longer time scales than a typical duration of a single nerve signal pulse. From that perspective, a more accurate representation of a cytoskeleton in the mathematical models could allow one to describe causal connections that might be missed in a more abstract description where the influence of the cytoskeleton, even if taken into account, is hidden away in some static parameter affecting a single aspect of the model. Unfortunately, as noted in the previous chapter, more experimental observations would be needed. It is not impossible that after more detailed experimental work emerges the cytoskeleton could be considered as important for the signal propagation in nerves as the cell membrane is, as an example.

Thirdly, the interdisciplinarity also may help in building the mathematical models. For example, the description of nonlinear fractional waves in phospholipid monolayers (Kappler et al., 2017) may be used for modeling the processes in biomembranes as a transition between the hyperbolic (wave-type) and parabolic (diffusive-type) behaviors. The concept of internal variables may beside the description of exo- and endothermic reactions also used for more accurately describing the influence of the cytoskeleton on the mechanical properties (membrane stiffness, axoplasm viscosity) or small scale geometry (for example, myelin distribution along the axon). In the classical HH model the phenomenological variables used for ionic currents are, in essence, internal variables describing the kinematics of “hidden” physical structure in the form of total summarized effect of various individual ionic channels and their opening and closing through their effect on the macroscopic/observable process which is the AP. Another aspect that could benefit from additional clarification in the mathematical models is the role of chemistry in the process of nervous pulse propagation. However, here one should be careful by keeping in mind the time and space scales of the problem, diffusion, in general, is relatively slow, however, over short enough distances both chemistry and diffusion could be fast enough to influence the dynamics of nerve signal propagation. And, obviously, the mathematical models constructed for describing idealized structures on which idealized nervous signals are propagating would need eventually a practical application in medicine which deals with real living systems where almost everything affects everything else so the constructed models need to be robust enough to be able to deal with all that somehow.

Fourthly, the general energy balance between the electrical, mechanical and thermal effects must be better explained. There are several studies where this problem is analyzed (de A. Nogueira and Conde Garcia, 1983; Mussel and Schneider, 2019; Engelbrecht et al., 2021; Peets et al., 2021) but the full analysis is still absent. In this context, an interesting question is whether the mechanisms of heat transfer will be better explained by modifications of Fourier's law like proposed for biological tissues by Shomali et al. (2022).

Toffler (1984) has noted once upon time that: “One of the most highly developed skills in contemporary Western civilization is dissection: the split-up of problems into their smallest possible components. We are good at it. So good, we often forget to put the pieces back together again.” So, we should do our best to fit the smaller, manageable, understandable details back together, figure out what is truly important and what is small enough to be safe to leave aside for the sake of simplicity and then, so to say, see the forest among all the trees. Understand how all the individual interesting details affect the larger picture.

## 6. Conclusions

We have demonstrated that for modeling signals in nerves, the physical considerations must be followed. Biological systems are extremely complicated and the governing equations for describing processes need modifications to reflect their behavior. It is said that “Newton rules biology” (Pennycuick, 1992) and “Mathematics is biology's next microscope” (Cohen, 2004) but experimental results must be carefully checked to estimate the correctness of models. The rules of modeling should follow simple principles: check the laws of physics, modify the constitutive relations (dependencies of variables) for grasping the measured effects, and remember that every change in a field variable will cause a change in other variables but the formalism (mathematics) must be related to physical mechanisms. The strength of mathematical modeling is related to the possibility to proceed with *in silico* experiments that also could help to determine the physical parameters in addition to experiments *in vivo* or *in vitro*.

## Author contributions

TP prepared the draft. KT prepared the figures. All authors contributed to the article and approved the submitted version.

## References

[B1] Abbott B. C. Hill A. V. Howarth J. V. (1958). The positive and negative heat production associated with a nerve impulse. Proc. R. Soc. B Biol. Sci. 148, 149–187. 10.1098/rspb.1958.0012 13518134

[B2] Ablowitz M. J. (2011). Nonlinear dispersive waves. Cambridge: Cambridge University Press.

[B3] Akkin T. Joo C. De Boer J. F. (2007). Depth-resolved measurement of transient structural changes during action potential propagation. Biophys. J. 93, 1347–1353. 10.1529/biophysj.106.091298 17526590 PMC1929037

[B4] Alvargonzález D. (2011). Multidisciplinarity, interdisciplinarity, transdisciplinarity, and the sciences. Int. Stud. Philos. Sci. 25, 387–403. 10.1080/02698595.2011.623366

[B5] Andersen S. S. Jackson A. D. Heimburg T. (2009). Towards a thermodynamic theory of nerve pulse propagation. Prog. Neurobiol. 88, 104–113. 10.1016/j.pneurobio.2009.03.002 19482227

[B6] Arancibia-Carcamo I. L. Attwell D. (2014). The node of Ranvier in CNS pathology. Acta Neuropathol. 128, 161–175. 10.1007/s00401-014-1305-z 24913350 PMC4102831

[B7] Berezovski A. (2020). “Elastic waves in microstructured solids,” in Encyclopedia of Continuum Mechanics, eds Altenbach H. Öchsner A. (Berlin, Heidelberg: Springer Berlin Heidelberg), 830–837.

[B8] Bressloff P. C. (2014). Waves in Neural Media. Lecture Notes on Mathematical Modelling in the Life Sciences (New York, NY: Springer).

[B9] Chen H. Garcia-Gonzalez D. Jérusalem A. (2019). Computational model of the mechanoelectrophysiological coupling in axons with application to neuromodulation. Phys. Rev. E 99, 032406. 10.1103/PhysRevE.99.032406 30999419

[B10] Clay J. R. (2005). Axonal excitability revisited. Prog. Biophys. Mol. Biol. 88, 59–90. 10.1016/j.pbiomolbio.2003.12.004 15561301

[B11] Cohen J. E. (2004). Mathematics is biology's next microscope, only better; biology is mathematics' next physics, only better. PLoS Biol. 2, e439. 10.1371/journal.pbio.0020439 15597117 PMC535574

[B12] Contera S. (2019). Nano Comes to Life: How Nanotechnology Is Transforming Medicine and the Future of Biology. Princeton; Oxford: Princeton University Press.

[B13] de A. Nogueira R. Conde Garcia E. (1983). A theoretical study on heat production in squid giant axon. J. Theor. Biol. 104, 43–52. 10.1016/0022-5193(83)90400-9 6314059

[B14] Debanne D. Campanac E. Bialowas A. Carlier E. Alcaraz G. (2011). Axon physiology. Physiol. Rev. 91, 555–602. 10.1152/physrev.00048.2009 21527732

[B15] Doyle D. A. Cabral J. M. Pfuetzner R. A. Kuo A. Gulbis J. M. Cohen S. L.. (1998). The structure of the potassium channel: molecular basis of K^+^ conduction and selectivity. Science 280, 69–77. 10.1126/science.280.5360.69 9525859

[B16] Drukarch B. Holland H. A. Velichkov M. Geurts J. J. Voorn P. Glas G.. (2018). Thinking about the nerve impulse: a critical analysis of the electricity-centered conception of nerve excitability. Prog. Neurobiol. 169, 172–185. 10.1016/j.pneurobio.2018.06.009 29981394

[B17] El Hady A. Machta B. B. (2015). Mechanical surface waves accompany action potential propagation. Nat. Commun. 6, 6697. 10.1038/ncomms7697 25819404

[B18] Engelbrecht J. (1981). On theory of pulse transmission in a nerve fibre. Proc. R. Soc. A Math. Phys. Eng. Sci. 375, 195–209. 10.1098/rspa.1981.0047

[B19] Engelbrecht J. (1991). Introduction to Asymmetric Solitary Waves. New York, NY: Longman Scientific & Technical and Wiley.

[B20] Engelbrecht J. Tamm K. Peets T. (2015). On mathematical modelling of solitary pulses in cylindrical biomembranes. Biomech. Model. Mechanobiol. 14, 159–167. 10.1007/s10237-014-0596-2 24848645

[B21] Engelbrecht J. Tamm K. Peets T. (2021). Modelling of Complex Signals in Nerves. Cham: Springer International Publishing.

[B22] Eringen A. C. (1962). Nonlinear Theory of Continuous Media. New York, NY: McGraw-Hill Book Company.

[B23] Eringen A. C. Maugin G. A. (1990). Electrodynamics of Continua I. New York, NY: Springer New York.

[B24] Ermentrout G. B. Terman D. H. (2010). Mathematical Foundations of Neuroscience, vol. 35 of Interdisciplinary Applied Mathematics. New York, NY: Springer.

[B25] Fields R. D. (2014). Myelin - More than insulation. Science 344, 264–266. 10.1126/science.1253851 24744365 PMC5017201

[B26] Franke T. Leirer C. Wixforth A. Schneider M. F. (2009). Phase transition induced adhesion of giant unilamellar vesicles. ChemPhysChem 10, 2858–2861. 10.1002/cphc.200800555 19598193

[B27] Giugliano M. Negrello M. Linaro D. (eds.) (2022). Computational Modelling of the Brain, vol. 1359 of Advances in Experimental Medicine and Biology. Cham: Springer International Publishing.

[B28] Gonzalez-Perez A. Mosgaard L. Budvytyte R. Villagran-Vargas E. Jackson A. Heimburg T. (2016). Solitary electromechanical pulses in lobster neurons. Biophys. Chem. 216, 51–59. 10.1016/j.bpc.2016.06.005 27448851

[B29] Goriely A. Geers M. G. Holzapfel G. A. Jayamohan J. Jérusalem A. Sivaloganathan S.. (2015). Mechanics of the brain: perspectives, challenges, and opportunities. Biomech. Model. Mechanobiol. 14, 931–965. 10.1007/s10237-015-0662-4 25716305 PMC4562999

[B30] Griesbauer J. Bössinger S. Wixforth A. Schneider M. F. (2012). Propagation of 2D pressure pulses in lipid monolayers and its possible implications for biology. Phys. Rev. Lett. 108, 198103. 10.1103/PhysRevLett.108.198103 23003093

[B31] Hall C. W. (1999). Laws and Models: Science, Engineering, and Technology. Boca Raton: CRC Press.

[B32] Han K. S. Guo C. Chen C. H. Witter L. Osorno T. Regehr W. G. (2018). Ephaptic coupling promotes synchronous firing of cerebellar Purkinje cells. Neuron 100, 564–578.e3. 10.1016/j.neuron.2018.09.018 30293822 PMC7513896

[B33] Heimburg T. (2010). Lipid ion channels. Biophys. Chem. 150, 2–22. 10.1016/j.bpc.2010.02.018 20385440

[B34] Heimburg T. (2012). The capacitance and electromechanical coupling of lipid membranes close to transitions: the effect of electrostriction. Biophys. J. 103, 918–929. 10.1016/j.bpj.2012.07.010 23009841 PMC3433620

[B35] Heimburg T. Jackson A. D. (2005). On soliton propagation in biomembranes and nerves. Proc. Natl. Acad. Sci. U. S. A. 102, 9790–9795. 10.1073/pnas.0503823102 15994235 PMC1175000

[B36] Hines M. L. Carnevale N. T. (2001). NEURON: a tool for neuroscientists. Neuroscientist 7, 123–135. 10.1177/107385840100700207 11496923

[B37] Hodgkin A. L. Huxley A. F. (1952). A quantitative description of membrane current and its application to conduction and excitation in nerve. J. Physiol. 117, 500–544. 10.1113/jphysiol.1952.sp004764 12991237 PMC1392413

[B38] Holland L. de Regt H. W. Drukarch B. (2019). Thinking about the nerve impulse: the prospects for the development of a comprehensive account of nerve impulse propagation. Front. Cell. Neurosci. 13, 1–12. 10.3389/fncel.2019.00208 31156394 PMC6529593

[B39] Howarth J. V. Keynes R. D. Ritchie J. M. (1968). The origin of the initial heat associated with a single impulse in mammalian non-myelinated nerve fibres. J. Physiol. 194, 745–793. 10.1113/jphysiol.1968.sp008434 5636997 PMC1365662

[B40] Iwasa K. Tasaki I. Gibbons R. (1980). Swelling of nerve fibers associated with action potentials. Science 210, 338–339. 10.1126/science.7423196 7423196

[B41] Jerusalem A. Al-Rekabi Z. Chen H. Ercole A. Malboubi M. Tamayo-Elizalde M.. (2019). Electrophysiological-mechanical coupling in the neuronal membrane and its role in ultrasound neuromodulation and general anaesthesia. Acta Biomater. 97, 116–140. 10.1016/j.actbio.2019.07.041 31357005

[B42] Jérusalem A. García-Grajales J. A. Merchán-Pérez A. Peña J. M. (2014). A computational model coupling mechanics and electrophysiology in spinal cord injury. Biomech. Model. Mechanobiol. 13, 883–896. 10.1007/s10237-013-0543-7 24337934

[B43] Kappler J. Shrivastava S. Schneider M. F. Netz R. R. (2017). Nonlinear fractional waves at elastic interfaces. Phys. Rev. Fluids 2, 1–18. 10.1103/PhysRevFluids.2.114804

[B44] Kaufmann K. (1989). Action Potentials and Electromechanical Coupling in the Macroscopic Chiral Phospholipid Bilayer. Caruaru. Available online at: https://www.nbi.ku.dk/membranes/Kaufmann/pdf/1989_Kaufmann_book4_org.pdf

[B45] Ling T. Boyle K. C. Zuckerman V. Flores T. Ramakrishnan C. Deisseroth K.. (2020). High-speed interferometric imaging reveals dynamics of neuronal deformation during the action potential. Proc. Natl. Acad. Sci. U. S. A. 117, 10278–10285. 10.1073/pnas.1920039117 32341158 PMC7229674

[B46] Lodish H. Berk A. Matsudaira P. Kaiser C. A. Krieger M. Scott M. P. (2004). Transport of ions and small molecules across cell membranes. Mol. Cell Biol. 7, 245–300.

[B47] Lucht P. (2014). Transmission Lines and Maxwell's Equations. Salt Lake City: Rimrock Digital Technology.

[B48] Malischewsky P. (1987). Surface Waves and Discontinuities. Amsterdam: Elsevier.

[B49] Maugin G. A. (1990). Internal variables and dissipative structures. J. Nonequil. Thermodyn. 15, 173–192. 10.1515/jnet.1990.15.2.173

[B50] Maugin G. A. Engelbrecht J. (1994). A thermodynamical viewpoint on nerve pulse dynamics. J. Nonequil Thermodyn. 19, 9–23. 10.1515/jnet.1994.19.1.9

[B51] Maugin G. A. Muschik W. (1994). Thermodynamics with internal variables. Part I. General concepts. J. Nonequil. Thermodyn. 19, 217–249. 10.1515/jnet.1994.19.3.217

[B52] Mussel M. Schneider M. F. (2019). It sounds like an action potential: unification of electrical, chemical and mechanical aspects of acoustic pulses in lipids. J. R. Soc. Interface 16, 20180743. 10.1098/rsif.2018.0743 30958199 PMC6408356

[B53] Nagumo J. Arimoto S. Yoshizawa S. (1962). An active pulse transmission line simulating nerve axon. Proc. IRE 50, 2061–2070. 10.1109/JRPROC.1962.288235

[B54] National Research Council (2005). Catalyzing Inquiry at the Interface of Computing and Biology. Washington, DC: The National Academies Press. 20669460

[B55] Nelson P. C. Radosavljevic M. Bromberg S. (2003). Biological Physics: Energy, Information, Life. New York, NY: W.H. Freeman and Company.

[B56] Nicoletti M. Chiodo L. Loppini A. (2021). Biophysics and modeling of mechanotransduction in neurons: a review. Mathematics 9, 1–32. 10.3390/math904032336439246

[B57] Peets T. Tamm K. Engelbrecht J. (2021). On the physical background of nerve pulse propagation: heat and energy. J. Nonequil. Thermodyn. 46, 343–353. 10.1515/jnet-2021-0007

[B58] Peng H. Xie P. Liu L. Kuang X. Wang Y. Qu L.. (2021). Morphological diversity of single neurons in molecularly defined cell types. Nature 598, 174–181. 10.1038/s41586-021-03941-1 34616072 PMC8494643

[B59] Pennycuick C. J. (1992). Newton Rules Biology: A Physical Approach to Biological Problems Oxford: Oxford University Press.

[B60] Rall W. (1977). “Core conductor theory and cable properties of neurons,” in Comprehensive Physiology, Vol. 1, ed Kandel E. R. (Bethesda, MD: American Physiology Society), 39–97.

[B61] Rvachev M. M. (2010). On axoplasmic pressure waves and their possible role in nerve impulse propagation. Biophys. Rev. Lett. 5, 73–88. 10.1142/S1793048010001147

[B62] Schmidt H. Hahn G. Deco G. Knösche T. R. (2021). Ephaptic coupling in white matter fibre bundles modulates axonal transmission delays. PLoS Comput. Biol. 17, 1–24. 10.1371/journal.pcbi.1007858 33556058 PMC7895385

[B63] Schneider M. F. (2021). Living systems approached from physical principles. Prog. Biophys. Mol. Biol. 162, 2–25. 10.1016/j.pbiomolbio.2020.10.001 33068591

[B64] Scott A. (1999). Nonlinear Science. Emergence and Dynamics of Coherent Structures (Oxford: Oxford University Press).

[B65] Scott A. (2002). Neuroscience: A Mathematical Primer. New York, NY: Springer Science & Business Media.

[B66] Sherrington C. S. (1906). The Integrative Action of the Nervous System. New Haven, CT: Yale University Press.

[B67] Shomali Z. Kovács R. Ván P. Kudinov I. V. Ghazanfarian J. (2022). Lagging heat models in thermodynamics and bioheat transfer: a critical review. Contin. Mech. Thermodyn. 34, 637–679. 10.1007/s00161-022-01096-6

[B68] Singh P. Sahoo P. Saxena K. Manna J. S. Ray K. Ghosh S.. (2021). Cytoskeletal filaments deep inside a neuron are not silent: they regulate the precise timing of nerve spikes using a pair of vortices. Symmetry 13, 821. 10.3390/sym13050821

[B69] Slattery J. C. (1971). Momentum, Energy, and Mass Transfer in Continua. New York, NY: McGraw Hill.

[B70] Sula A. Booker J. Ng L. C. Naylor C. E. Decaen P. G. Wallace B. A. (2017). The complete structure of an activated open sodium channel. Nat. Commun. 8, 2–10. 10.1038/ncomms14205 28205548 PMC5316852

[B71] Tamm K. Engelbrecht J. Peets T. (2019). Temperature changes accompanying signal propagation in axons. J. Nonequil. Thermodyn. 44, 277–284. 10.1515/jnet-2019-0012

[B72] Tamm K. Peets T. Engelbrecht J. (2022). Mechanical waves in myelinated axons. Biomech. Model. Mechanobiol. 21, 1285–1297. 10.1007/s10237-022-01591-4 35704223

[B73] Tasaki I. (1988). A macromolecular approach to excitation phenomena: mechanical and thermal changes in nerve during excitation. Physiol. Chem. Phys. Med. 20, 251–268. 3076013

[B74] Tasaki I. Byrne P. M. (1992). Heat production associated with a propagated impulse in Bullfrog myelinated nerve fibers. Jpn. J. Physiol. 42, 805–813. 10.2170/jjphysiol.42.805 1491504

[B75] Tasaki I. Kusano K. Byrne P. M. (1989). Rapid mechanical and thermal changes in the garfish olfactory nerve associated with a propagated impulse. Biophys. J. 55, 1033–1040. 10.1016/S0006-3495(89)82902-9 2765644 PMC1330571

[B76] Terakawa S. (1985). Potential-dependent variations of the intracellular pressure in the intracellularly perfused squid giant axon. J. Physiol. 369, 229–248. 10.1113/jphysiol.1985.sp015898 4093881 PMC1192646

[B77] Tian J. Huang G. Lin M. Qiu J. Sha B. Lu T. J.. (2019). A mechanoelectrical coupling model of neurons under stretching. J. Mech. Behav. Biomed. Mater. 93, 213–221. 10.1016/j.jmbbm.2019.02.007 30826698

[B78] Toffler A. (1984). Foreword to: Order Out of Chaos: Man's New Dialogue with Nature. London: Heinemann.

[B79] Watanabe A. (1986). Mechanical, thermal, and optical changes of the nerve membrane associated with excitation. Jpn. J. Physiol. 36, 625–643. 10.2170/jjphysiol.36.625 3537414

